# Cluster features in fibrosing interstitial lung disease and associations with prognosis

**DOI:** 10.1186/s12890-023-02735-7

**Published:** 2023-11-01

**Authors:** Yuanying Wang, Di Sun, Jingwei Wang, Shiwen Yu, Na Wu, Qiao Ye

**Affiliations:** 1 Clinical Center for Interstitial Lung Diseases, Beijing Institute of Respiratory Medicine, Capital Medical University Affiliated Beijing Chao-Yang Hospital, Beijing, China; 2 Department of Occupational Medicine and Toxicology, Capital Medical University Affiliated Beijing Chao-Yang Hospital, Beijing, China

**Keywords:** Pulmonary fibrosis, Interstitial lung diseases, Cluster analysis, Prognosis, Acute exacerbation

## Abstract

**Background:**

Clustering is helpful in identifying subtypes in complex fibrosing interstitial lung disease (F-ILD) and associating them with prognosis at an early stage of the disease to improve treatment management. We aimed to identify associations between clinical characteristics and outcomes in patients with F-ILD.

**Methods:**

Retrospectively, 575 out of 926 patients with F-ILD were eligible for analysis. Four clusters were identified based on baseline data using cluster analysis. The clinical characteristics and outcomes were compared among the groups.

**Results:**

Cluster 1 was characterized by a high prevalence of comorbidities and hypoxemia at rest, with the worst lung function at baseline; Cluster 2 by young female patients with less or no smoking history; Cluster 3 by male patients with highest smoking history, the most noticeable signs of velcro crackles and clubbing of fingers, and the severe lung involvement on chest image; Cluster 4 by male patients with a high percentage of occupational or environmental exposure. Clusters 1 (median overall survival [OS] = 7.0 years) and 3 (OS = 5.9 years) had shorter OS than Clusters 2 (OS = not reached, Cluster 1: p < 0.001, Cluster 3: p < 0.001) and 4 (OS = not reached, Cluster 1: p = 0.004, Cluster 3: p < 0.001). Clusters 1 and 3 had a higher cumulative incidence of acute exacerbation than Clusters 2 (Cluster 1: p < 0.001, Cluster 3: p = 0.014) and 4 (Cluster 1: p < 0.001, Cluster 3: p = 0.006). Stratification by using clusters also independently predicted acute exacerbation (p < 0.001) and overall survival (p < 0.001).

**Conclusions:**

The high degree of disease heterogeneity of F-ILD can be underscored by four clusters based on clinical characteristics, which may be helpful in predicting the risk of fibrosis progression, acute exacerbation and overall survival.

**Supplementary Information:**

The online version contains supplementary material available at 10.1186/s12890-023-02735-7.

## Introduction

Fibrosing interstitial lung disease (F-ILD) encompasses a heterogeneous group of diseases of various etiology, including idiopathic pulmonary fibrosis (IPF), connective tissue disease-associated interstitial lung disease (CTD-ILD), idiopathic non-specific interstitial pneumonia (iNSIP), fibrotic hypersensitivity pneumonitis (FHP); unclassifiable idiopathic interstitial pneumonia (uIIP), and occupational induced ILDs [1, 2]. Accurate diagnosis is essential for these diseases to management. However, a highly heterogeneous disease course often delays correct diagnosis and anti-fibrotic treatment in clinical practice.

Researchers have tried to find patients with irreversible rapidly progressive fibrosis, which may indicate higher mortality [3]. The term “progressive fibrosing ILD (PF-ILD)” used in a hallmark clinical trial was proposed to cover several diseases featuring high-resolution computed tomography (HRCT)-documented increase in the extent of pulmonary fibrosis, a decline in lung function, worsening respiratory symptoms, and a high risk of early mortality despite available treatments, with a clinical course similar to that of IPF [1, 2, 4, 5]. Recently, the newly updated American Thoracic Society (ATS)/European Respiratory Society (ERS)/Japanese Respiratory Society (JRS)/Asociación Latinoamericana de Tórax (ALAT) guideline suggests using the term “progressive pulmonary fibrosis (PPF)” instead. Still, the criteria of PF-ILD and PPF are different [6]. It is difficult to predict the proportion of patients with non-IPF ILDs who will develop a progressive fibrotic pattern; however, the likely predictors at high risk of progression and mortality at baseline have been investigated, such as male sex, increasing age, smoking, and HRCT-documented usual interstitial pneumonia (UIP) pattern [2, 3, 5, 7–9].

Using the baseline clinical features, a new trend of clustering method was aimed to help reveal a novel subtype of F-ILD, which was associated with the prognosis and treatment and had distinct clinical features. Patients with similar characteristics are grouped in the same cluster so that each cluster will be the most homogeneous and heterogeneous compared to the other clusters [10]. Previously, in chronic ILDs, clinical characteristics determined using cluster analysis illustrated considerable predictive accuracy for clinical outcomes, including progression-free survival, transplant-free survival, and acute exacerbation (AE) [11]. However, they didn’t include patients with occupational or environmental related ILDs, though occupational and environmental dust exposure is essential for clustering. Besides, the possible relationship between clusters and fibrosis progression has not been explored.

To elucidate potential relationships between clusters and outcomes, including fibrosis progression, AE, and all-cause mortality, we aimed to employ cluster analysis on a patient cohort obtained from a regional tertiary referral center specializing in the management of ILDs. We also conducted a comparison of the clinical characteristics among patients who met different criteria for fibrosis progression.

## Methods

### Study population and design

Patients with F-ILD aged ≥ 18 years between January 1, 2016, and January 1, 2021, from Beijing Chao-Yang Hospital, China, were retrospectively screened for the study. The diagnoses were based on multidisciplinary discussion, following ATS/ ERS guidelines [12, 13]. Patients enrolled in the study were those whose baseline medical records, pulmonary function tests, and HRCT findings were accessible, and who had at least two documented follow-up visits with follow-up intervals of less than one year. Following were the exclusion criteria: (1) < 10% fibrosis documented on baseline HRCT, (2) diagnosis of pulmonary embolism, decompensated heart failure, lower respiratory tract infections, or acute respiratory distress syndrome at baseline, (3) lung cancer at baseline, (4) missing available baseline data, (5) loss to follow-up (**Supplemental Material Figure ****S1**). The study was approved by the Ethics Committee of Beijing Chao-Yang Hospital (2020-KY-437) and performed by the principles of the Declaration of Helsinki.

### Data collection

The electronic medical record was reviewed to extract pertinent data at the first visit, including age, sex, body mass index (BMI), symptoms, signs, smoking history, occupational and environmental exposures based on each patient’s reported history, comorbidities (pulmonary hypertension [defined by the right heart catheterization demonstrating a mean pulmonary artery pressure greater than 20 mmHg], diabetes, coronary heart disease, hypertension, hypothyroidism, gastroesophageal reflux disease [GERD]). All patients provided laboratory test results, pulmonary function measures [forced vital capacity (FVC), percent predicted for FVC (FVC% pred), and percent predicted for diffusion capacity of the lung for carbon monoxide (DLCO% pred)] and HRCT images. Laboratory data included arterial blood gas test results, antinuclear antibody (ANA) titers, routine blood counts, the derivative blood cell count inflammation indexes (neutrophil-to-lymphocyte ratio, monocyte-to-lymphocyte ratio, platelet-to-lymphocyte ratio), and serum oncomarkers (squamous cell carcinoma [SCC] antigen, carcinoembryonic antigen [CEA], cytokeratin fraction 21–1[CYFRA21-1], carbohydrate antigen 125 [CA125], and neuron-specific enolase [NSE]). Hypoxemia was defined as a partial pressure of oxygen in the arterial blood of less than 80 mm Hg obtained from an arterial blood gas test at rest. Two radiologists blinded to the clinical data independently reviewed the HRCT scans. The disagreements were resolved via consensus. An HRCT-documented UIP-like pattern was considered a definitive UIP or probable UIP pattern according to the Clinical Practice Guideline of IPF [6, 12].

We reviewed each case to gather treatment options. The choice of treatment depended on the decision of physicians and the use of each treatment was documented if it was ever used for at least one month. The treatment regimens for patients consisted of corticosteroids, both in monotherapy and in combination with immunosuppressive drugs (cyclophosphamide, mycophenolate mofetil, cyclosporine, azathioprine or tacrolimus), antifibrotic therapy (nintedanib or pirfenidone), and long-term oxygen therapy (at least 15 h per day). Each patient’s Gender-Age-Physiology (GAP) score was calculated and assigned to their respective GAP stage. The GAP index was preferred over the ILD-GAP index, as some patients had ILD diagnoses that did not align with the ILD categories in the ILD-GAP index [14, 15].

### Follow-up and outcomes of the study

Follow-up data were obtained from outpatient follow-up records (usually every 3–6 months), hospitalization details, and telephone interviews and the follow-up period ended on January 1, 2022. The outcome of the study was the (1) occurrence of fibrosis progression, (2) occurrence of AE, and (3) all-cause mortality. AE was defined as an acute, clinically significant respiratory deterioration that occurred in less than one month and was accompanied by new radiologic abnormalities on HRCT, such as diffuse, bilateral ground-glass opacification, with no obvious clinical cause, such as fluid overload, left heart failure, or pulmonary embolism [16, 17]. Overall survival (OS) was measured from the first visit to death from any cause.

### Fibrosis progression

Both two different definitions explain fibrosis progression in the cohort. Firstly, PPF was defined as at least two of three criteria (worsening symptoms, radiological progression, and physiological progression) occurring within 12 months (Criteria A) [6]. Physiological progression was defined as either of the following: (1) absolute decline in FVC% pred > 5%; (2) absolute decline in DLCO% pred > 10% within 12 months of follow-up [6]. Notably, our cohort included patients with IPF. Secondly, Patients meeting any of the following criteria within 24 months period have experienced fibrosis progression (Criteria B): (a) a relative decline of ≥ 10% in FVC% pred; (b) a relative decline of ≥ 15% in DLCO% pred; (c) worsening symptoms or a worsening radiological appearance accompanied by a ≥ 5–<10% relative decrease in FVC% pred [1].

### Statistical analysis

Statistical analyses were performed using the SPSS Statistics software, version 26 (IBM, Inc., Chicago, IL, USA). After excluding those with missing baseline data, the complete data of 575 patients were used for cluster analysis. Based on previous literature, 17 variables were identified with substantial clinical relevance for inclusion in the “two-step” cluster analysis [1, 11, 18]. These variables were as follows: male, age, BMI, heavy smoking, exposure history, hypoxemia, signs [velcro crackles, clubbing of fingers], lung function [FVC, FVC% pred, DLCO% pred], HRCT features [UIP-like pattern, emphysema], ANA titers and comorbidities [pulmonary hypertension, diabetes, coronary heart disease]). The choice of a similarity measure was based on the log-likelihood distance. To determine which number of clusters is “best”, each of the cluster solutions was compared using Schwarz’s Bayesian Criterion as the clustering criterion. The optimal number of four clusters was automatically determined. The silhouette coefficient was 0.5 and the cluster quality was fair. When we specified the number at three or five, the silhouette coefficient was lower than 0.5. The discriminant function analysis was performed to validate the cluster analysis results, and 93.7% of original grouped cases were correctly classified.

Data were expressed as means (standard deviation, SD) or medians (quartile) depending on distribution and numbers (percentage), respectively. Mann–Whitney U, Kruskal–Wallis test, or One-way ANOVA was used for continuous variables and the chi-squared test or Fisher’s exact test for categorical variables with Bonferroni post-hoc tests to compare the difference between every two groups. Survival curves were obtained using the Kaplan-Meier method. Survival was assessed using unadjusted log-rank testing and univariate and multivariable Cox proportional hazards regression. All statistical tests were 2-sided, and a p-value < 0.05 was considered statistically significant.

## Results

### Demographic and clinical characteristics of the whole cohort

The final cohort included 575 patients with 3.5 years median follow-up time. A summary of the demographic and clinical characteristics of all the patients included in this cohort was shown in Table 1. 94 (16.3%), 299 (52%), 2 (0.3%), 48 (8.3%), 36 (6.3%), 93 (16.2%), and 3(1.8%) patients were diagnosed with IPF, CTD-ILD, iNSIP, FHP, uIIP, occupational induced ILDs (asbestos/chronic silicosis) and sarcoidosis, respectively (Table 2).

**Table 1:** Demographics and clinical characteristics of the patients in four clusters

		Cluster 1, n = 181	Cluster 2, n = 164	Cluster 3, n = 134	Cluster 4, n = 96	All, n = 575	P
#Age (quartile)		65 (57,71)^†^	59.5 (49,68.5)^‡§^	66 (60,73)^§^	62 (59,69)	63 (56,70)	< 0.001
#Male, n (%)		57 (51.5)^†‡*^	30 (18.3)^‡§^	119 (88.8)	87 (90.6)	293 (51.0)	< 0.001
#BMI (quartile)		25.9 (24.1,28.4)	25.7 (23.9,27.5)	25.8 (23.6,27.5)	25.9 (24.1,28.2)	25.9 (23.9,27.9)	0.351
Smoking status, n (%)							
	Ever smokers	31 (17.1)^‡§^	18 (11.0)^‡§^	107 (79.9)	62 (64.6)	218 (37.9)	< 0.001
	#Heavy smokers*	12 (6.6)^‡§^	6 (3.7)^‡§^	91 (67.9)	51 (53.1)	160 (27.8)	< 0.001
	Smoking cessation > 1 yr	24 (13.3)^‡^	16 (9.8)	77 (57.5)	40 (41.7)	157 (27.3)	0.003
#Exposure history, n (%)		73 (40.3)^§^	46 (28.0)	38 (28.4)	84 (87.5)	241 (41.9)	< 0.001
	Inorganic	48 (26.5)^†‡§^	12 (7.3)^§^	18 (13.4)^§^	51 (53.1)	129 (22.4)	
	Organic	20 (11.0)^§^	31 (18.9)	16 (11.9)^§^	25 (26.0)	92 (16.0)	
	Mixed	4 (2.2)	3 (1.8)	3 (2.2)	6 (6.3)	16 (2.8)	< 0.001
Symptoms, n (%)							
	Dry cough	127 (90.7)^†^	88 (77.2)	85 (82.5)	46 (82.1)	346 (83.8)	0.032
	Productive cough	44 (24.3)^†^	20 (12.2)	15 (11.2)	19 (19.8)	98 (17.0)	0.004
	Shortness of breath	126 (69.6)^†§^	80 (48.8)	83 (61.9)^§^	34 (35.4)	323 (56.2)	< 0.001
	Joint discomfort	37 (20.4)	39 (23.8)^‡§^	15 (11.2)	9 (9.4)	100 (17.4)	0.003
Signs, n (%)							
	#Clubbing of fingers	37 (20.4)^‡§^	46 (28.0)^‡§^	74 (55.2)^§^	1 (1.0)	158 (27.5)	< 0.001
	#Velcro crackles	137 (75.7)^‡§^	124 (75.6)^‡§^	128 (95.5)^§^	28 (29.2)	417 (72.5)	< 0.001
Pulmonary function							
	#FVC (L) (SD)	2.1 (0.64)	2.4 (0.7)	3.0 (0.7)	3.1 (0.9)	2.6 (0.8)	< 0.001
	#FVC% pred (quartile)	79.7(65.7,95.3)^†§^	89.6 (72.3,106.6)	85.1 (73.9,99.7)	87.3 (74.4,106.1)	85.2 (72,100.4)	< 0.001
	#DLCO% pred (quartile)	56.1 (45.2,71.2)^§^	61 (48.8,74)^§^	58 (44.2,70.5)^§^	71.7 (58.4,88)	60.3 (48.2,74)	< 0.001
HRCT, n (%)							
	#UIP-like pattern	21 (11.6)^†‡§^	15 (9.1)^‡^	99 (73.9)^§^	2 (2.1)	137 (23.8)	< 0.001
	#Emphysema	2 (1.1)^‡§^	0 (0)^‡§^	22 (16.4)	11 (11.5)	35 (6.1)	< 0.001
#Hypoxemia at rest, n (%)		120 (66.3)^†‡§^	0 (0)^‡§^	44 (32.8)^§^	11 (11.5)	175 (30.4)	< 0.001
#ANA, n (%)		97 (53.6)^‡§^	89 (54.3)	44 (32.8)^§^	12 (12.5)	242 (42.1)	< 0.001
Comorbidities, n (%)							
	#Pulmonary hypertension	45 (24.9)^†‡§^	5 (3.0)	13 (9.7)	4 (4.2)	67 (11.7)	< 0.001
	#Diabetes	91 (50.3)^†‡§^	0 (0)^‡§^	41 (30.6)^§^	11 (11.5)	143 (24.9)	< 0.001
	#Coronary heart disease	55 (30.4)^†‡^	5 (3.0)^‡§^	17 (12.7)	19 (19.8)	96 (16.7)	< 0.001
	Hypertension	88 (48.6)^†‡^	45 (27.4)	39 (29.1)	46 (47.9)	218 (37.9)	< 0.001
	Hypothyroidism	7 (3.9)	9 (5.5)^‡^	0 (0)	0 (0)	16 (2.8)	0.008
	GERD	43 (32.7)^§^	32 (29.7)	20 (24.2)	9 (17.4)	104 (18.1)	0.019
GAP-index							
	Score	3 (2,4)^†^	2 (1,3)^‡§^	3 (2,4)	3 (2,4)	3 (1,3)	< 0.001
	Stage I	134 (74.0)	152 (92.7)	93 (69.4)	68 (70.8)	447 (77.7)	
	Stage II	43 (23.7)	12 (7.3)	35 (26.1)	28 (29.2)	118 (20.5)	
	Stage III	4 (2.2)	0	6 (4.5)	0	10 (1.7)	< 0.001
Treatment, n (%)							
	Corticosteroid	133 (73.5)^‡§^	128 (78.0)^‡§^	56 (41.8)	41 (42.7)	358 (62.3)	< 0.001
	Immunosuppressive agents	97 (53.6)^‡§^	101 (61.6)^‡§^	33 (24.6)	20 (20.8)	251 (43.7)	< 0.001
	Anti-fibrotic treatment	38 (21.0)^†‡§^	25 (15.2)^‡^	51 (38.1)^§^	8 (8.3)	122 (21.2)	< 0.001
	Long-term oxygen therapy	34 (18.8)^†§^	12 (7.3)	21 (15.7)^§^	4 (4.2)	71 (12.3)	0.015

Data were presented as the mean (SD), median (quartile), or numbers (%)

#: Variables used for the clustering

*: Heavy smokers: smoking index = daily tobacco intake × duration of smoking ≥ 400

†: p < 0.05 compared with Cluster 2

‡: p < 0.05 compared with Cluster 3

§: p < 0.05 compared with Cluster 4

Abbreviations: ANA: antinuclear antibody; BMI: body-mass index; DLCO: diffusion capacity of the lung for carbon monoxide; FVC: forced vital capacity; GAP: genderage-physiology; GERD: gastro-esophageal reflux disease; HRCT: high-resolution computed tomography; SD: standard deviation; UIP: usual interstitial pneumonia

**Table 2:** Distinct diagnosis of the enrolled patients

	Cluster 1, n = 181	Cluster 2, n = 164	Cluster 3, n = 134	Cluster 4, n = 96	All, n = 575
IPF	8 (4.4)	3 (1.8)	76 (56.7)	7 (7.3)	94 (16.3)
CTD-ILD	120 (66.3)	119 (72.6)	42 (31.3)	18 (18.8)	299 (52.0)
iNSIP	1 (0.6)	1 (0.6)	0 (0)	0 (0)	2 (0.3)
FHP	7 (3.9)	20 (12.2)	6 (4.5)	15 (15.6)	48 (8.3)
uIIP	7 (3.9)	12 (7.3)	6 (4.5)	11 (11.5)	36 (6.3)
Occupational related ILDs	38 (21.0)	6 (3.7)	4 (3.0)	45 (46.9)	93 (16.2)
Chronic silicosis	4 (2.2)	1 (0.7)	0	12 (12.5)	17 (3.0)
Asbestos	34 (18.8)	5 (3.0)	4 (3.0)	33 (34.4)	76 (13.2)
Sarcoidosis	0 (0)	3 (1.8)	0 (0)	0 (0)	3 (1.8)

Data were expressed as numbers (%)

Abbreviations: CTD: connective tissue disease-associated; IPF: idiopathic pulmonary fibrosis; iNSIP: idiopathic non-specific interstitial pneumonia; FHP: fibrotic hypersensitivity pneumonitis; uIIP: unclassifiable idiopathic interstitial pneumonia; ILD: interstitial lung disease

### Clinical characteristics of the clusters

Seventeen variables were selected to apply the two-step method for clustering. A 4-cluster model best fit the overall dataset.

**Fig. 1:**
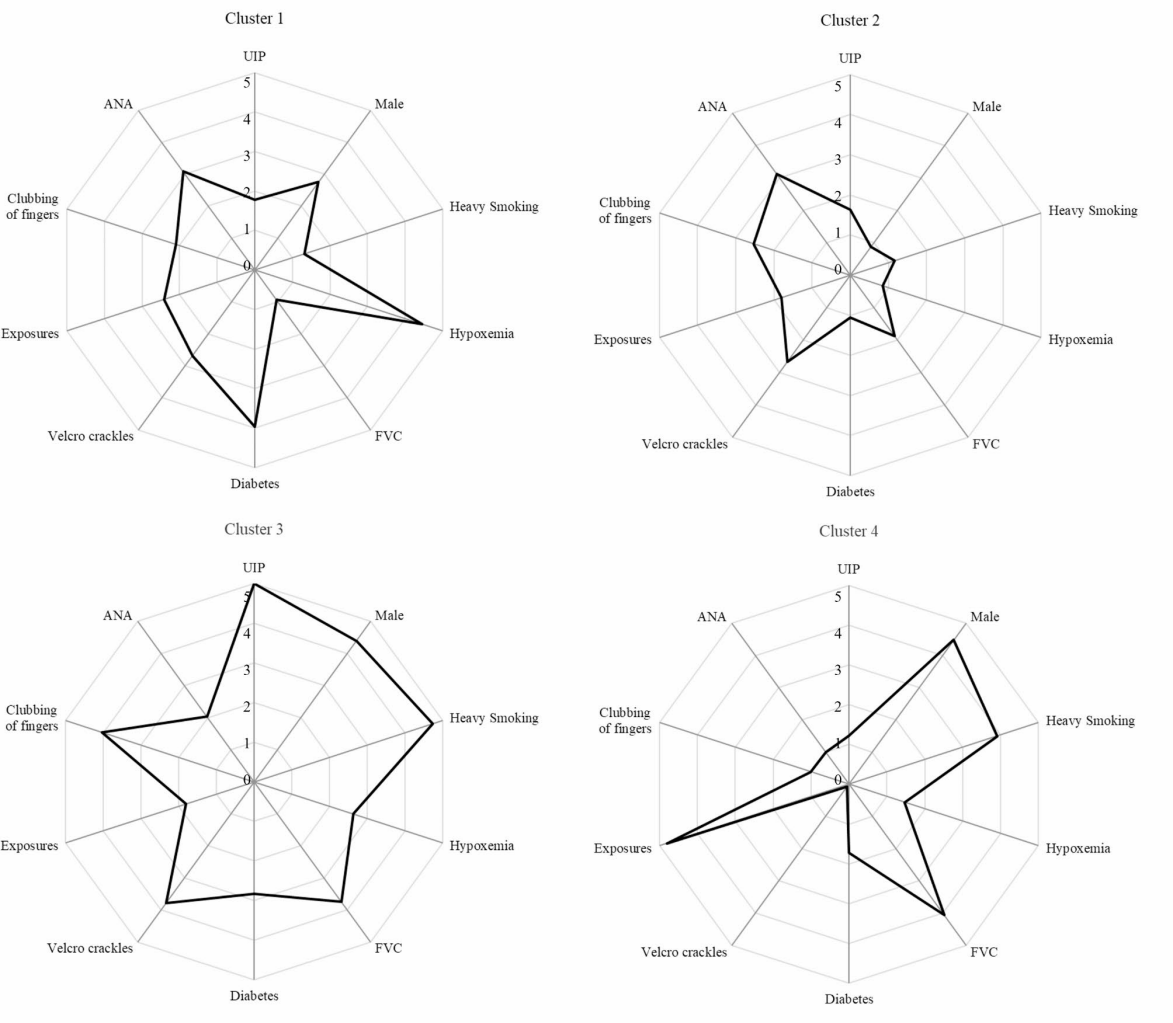
Radar plot of top 10 important predictors. Plot scale: 1, 0.6 standard deviations (SDs) below the mean; 2, 0.2 SDs below the mean; 3, > 0.2 SDs above the mean; 4, > 0.6 SDs above the mean; 5, > 1 SDs above the mean; Variables were displayed clockwise based on predictor’s importance. Abbreviations: ANA: antinuclear antibody; DLCO: diffusion capacity of the lung for carbon monoxide; FVC: forced vital capacity; UIP: usual interstitial pneumonia

Cluster 1 (n = 181) featured a higher prevalence of hypoxemia at rest (66.3%), lowest median FVC% pred (79.7%) and diabetes (50.3%) (Fig. 1; Table 1). The median age of patients was 65 years with almost equal distributions of males (51.5%) and females (48.5%). The cluster was with the second-lowest rate of smoking (17.1%) or heavy smoking (13.3%), and 53.6% of patients had autoimmune features (ANA > 1:100). Cluster 1 had the highest expression of white blood cell (WBC, 8.1 × 10^9^), neutrophils (5.7 × 10^9^), platelet (241.7 × 10^9^), neutrophil-to-lymphocyte ratio (4.8), platelet-to-lymphocyte ratio (180.7), and the highest expression of SCC (1.3 ng/ml), CYFRA21-1 (5.6 ng/ml), and NSE (17.2 ng/ml) (**Table ****S2**).

Cluster 2 (n = 164) described a cohort of predominantly female patients (81.7%) with the second-lowest rate of UIP-like fibrotic pattern (9.1%), least heavy smoking history (3.7%), and no hypoxemia was found (Fig. 1; Table 1). The median age was the youngest (59.5 years). The positive rate of ANA (ANA > 1:100) was the highest (54.6%). No emphysema was found in this cluster. It featured preserved FVC% pred (89.6%) and DLCO% pred (61%), the lowest prevalence of comorbidities. Regarding routine blood counts (**Table ****S2**), Cluster 2 had the lowest expression of WBC (6.7 × 10^9^), neutrophils (4.3 × 10^9^), lymphocytes (1.7 × 10^9^), monocyte (0.4 × 10^9^), and hemoglobin (125.0 g/L), and the lowest expression of SCC (0.9 ng/ml), CEA (2.2 ng/ml), and CA125 (16.4 U/ml).

Cluster 3 (n = 134) included a cohort of male patients (88.8%) with the highest rate of smoking (79.9%) or heavy smoking (67.9%), UIP-like fibrotic pattern (73.9%) on HRCT (Fig. 1; Table 1). The median age was 66 years and the percentages of clubbing of fingers (55.2%) and velcro crackles (95.5%) were the highest. The FVC% pred (85.1%) was preserved, while the DLCO% pred (58%) was the lowest. Regarding laboratory data (**Table ****S2**), it had the highest expression of monocyte (0.5 × 10^9^), hemoglobin (141.1 g/L), red cell distribution width (coefficient of variation) (15.9%), SCC (1.3 ng/ml), CEA (3.9 ng/ml), CYFRA21-1 (4.7 ng/ml) and CA125 (29.9 U/ml).

Cluster 4 (n = 96) featured the least UIP-like fibrotic pattern (2.1%), highest proportion of male patients (90.6%), and the most frequent exposure history (87.5%) (Fig. 1; Table 1**)**. The clinical signs of clubbing of fingers (1%) and velcro crackles (29.2%) and emphysema (11.5%) on HRCT were presented least (Table 1). The median FVC% pred was 87.3% and DLCO% pred was 71.1%. The positive rate of ANA (ANA > 1:100) was the lowest (12.5%) and it had the highest expression of monocyte-to-lymphocyte ratio (4.9) and lowest of platelet-to-lymphocyte ratio (122.7) (**Table ****S2**).

To assess baseline disease severity among the clusters, we evaluated the GAP score (Table 1). The median GAP score was similar among Cluster 1, Cluster 3 and Cluster 4 (GAP = 3). Cluster 2 had the lowest median score compared with others (GAP = 2, p < 0.001).

### Clinical outcomes among the clusters

**Fig. 2:**
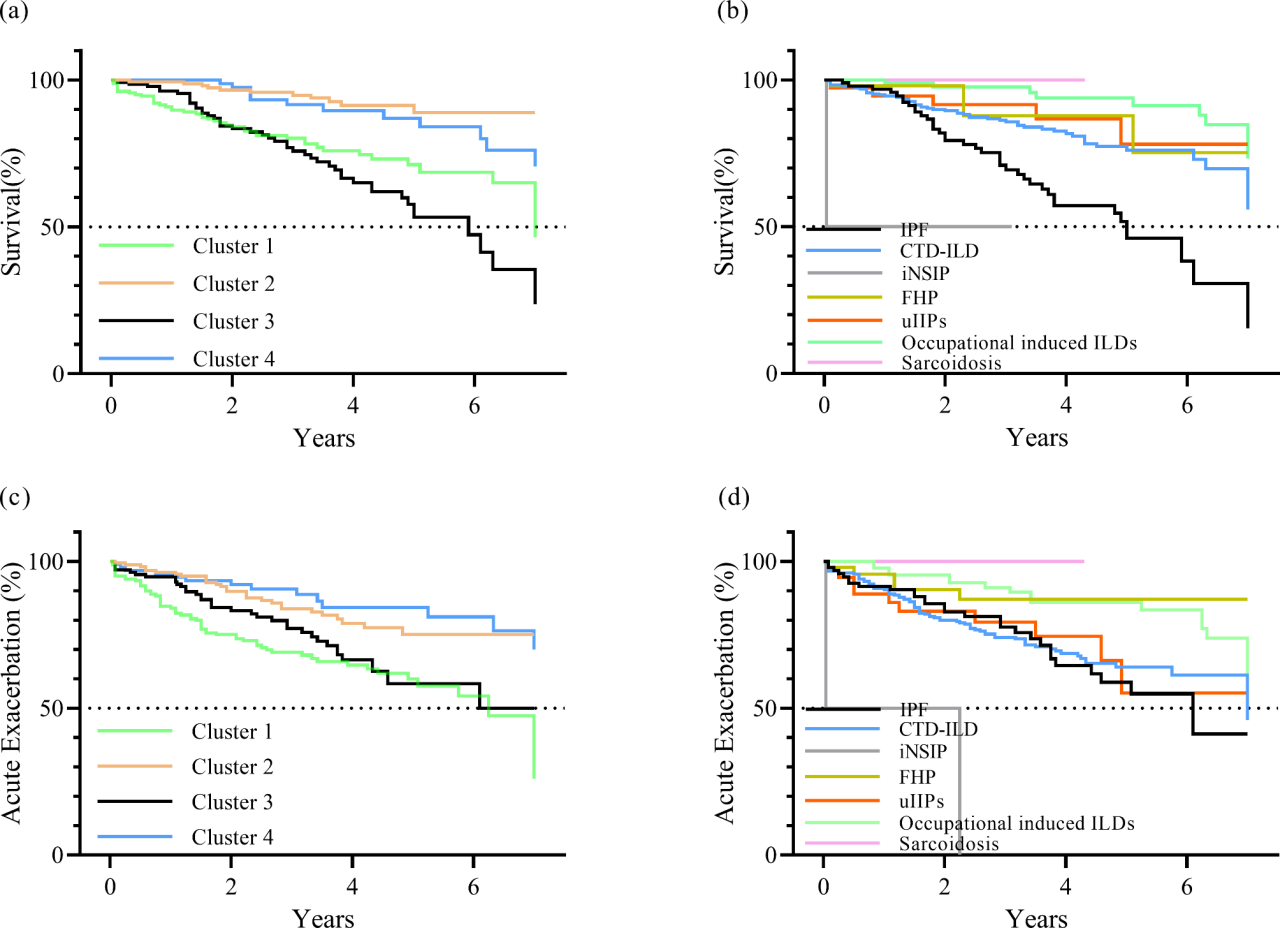
Overall survival (OS) and acute exacerbation (AE). (a) OS in 4 clusters. (b) OS in distinct diagnosis. (c) AE in 4 clusters. (d) AE in distinct diagnosis. Abbreviations: CTD: connective tissue disease; FHP: fibrotic hypersensitivity pneumonitis; ILD: interstitial lung disease; iNSIP: idiopathic non-specific interstitial pneumonia; IPF: idiopathic pulmonary fibrosis; uIIP: unclassifiable idiopathic interstitial pneumonia

The clinical outcomes of 4 clusters at the end of the study were shown in Table 3. The percentage for AE and mortality of the 4 clusters was 37%, 17.1%, 27.6%, 14.6%, and 24.9%, 6.7%, 33.6%, 12.5%, respectively. OS was significantly shorter in Cluster 1 (median OS = 7.0 years) and Cluster 3 (median OS = 5.9 years) than in Cluster 2 (Cluster 1: p < 0.001, Cluster 3: p < 0.001) and Cluster 4 (Cluster 1: p = 0.004, Cluster 3: p < 0.001) (Fig. 2**)**. The accumulative incidence rate of AE was higher in Clusters 1 and 3 than in Clusters 2 (Cluster 1: p < 0.001, Cluster 3: p = 0.014) and 4 (Cluster 1: p < 0.001, Cluster 3: p = 0.006). When stratified by diagnosis, OS was significantly shorter in patients with IPF (median OS = 5.0 years) than in CTD-ILD (p < 0.001), FHP (p = 0.004), and occupational induced ILDs (p < 0.001). Patients with IPF (p = 0.04) and (p = 0.043) had higher cumulative incidence rates of AE than FHP and patients with CTD-ILD had higher incidence rates than occupational induced ILDs (p = 0.003). Patients with uIIPs also had higher cumulative incidence rates than occupational-induced ILDs (p = 0.028). OS was shortest in patients with iNSIP than in others (p < 0.001), but only two patients were included in this group. Cluster 1 (Criteria A: 29.8%, Criteria B: 47%) and Cluster 3 (Criteria A: 35.8%, Criteria B: 49.3%) showed a higher rate of fibrosis progression despite different criteria for progression (Table 3). In addition, 150 out of 575 (26.1%) patients met Criteria A of fibrosis progression while 239 (41.6%) met Criteria B. Statistical significance was not observed in routine blood counts, oncomarkers and outcomes between patients with different criteria (**Tables ****S1**** and ****S3)**.

**Table 3:** Clinical outcomes of the patients in four clusters

	Cluster 1, n = 181	Cluster 2, n = 164	Cluster 3, n = 134	Cluster 4, n = 96	All, n = 575	P
Fibrosis progression, n (%)						
Criteria A (1 year), n (%)	54 (29.8)	32 (19.5)^‡^	48 (35.8)^§^	16 (16.7)	150 (26.1)	0.001
1	33 (18.2)	27 (16.5)	25 (18.7)	12 (12.5)	97 (16.9)	0.600
2	23 (12.7)	18 (11.0)	28 (20.9)	14 (14.6)	83 (14.4)	0.087
3	56 (30.9)	45 (27.4)	47 (35.1)^§^	18 (18.8)	166 (28.9)	0.049
4	79 (43.6)^†^	48 (29.3)	57 (42.5)	28 (29.2)	212 (36.9)	0.008
Criteria B (2 years), n (%)	85 (47.0)^§^	62 (37.8)	66 (49.3)^§^	26 (27.1)	239 (41.6)	0.002
a	23 (12.7)	21 (12.8)^‡^	16 (11.9)	7 (7.3)	67 (11.7)	0.534
b	29 (16.0)	14 (8.5)	27 (20.1)^§^	10 (10.4)	80 (13.9)	0.019
c	49 (27.1)^§^	39 (23.8)	42 (31.3)^§^	11 (11.5)	141 (24.5)	0.005
Acute exacerbation, n (%)	67 (37.0)^†‡§^	28 (17.1)^‡^	37 (27.6)^§^	14 (14.6)	146 (25.4)	< 0.001
Death, n (%)	45 (24.9)^†§^	11 (6.7)^‡^	45 (33.6)^§^	12 (12.5)	113 (19.7)	< 0.001

Data were presented as numbers (%)

†: p < 0.05 compared with Cluster 2

‡: p < 0.05 compared with Cluster 3

§: p < 0.05 compared with Cluster 4

Criteria A: at least two of three criteria (worsening symptoms, radiological progression, and physiological progression) as mentioned earlier. (1) absolute decline in percent predicted for forced vital capacity (FVC% pred) > 5%; (2) absolute decline in percent predicted for diffusion capacity of the lung for carbon monoxide (DLCO% pred) > 10%; (3) radiological progression; (4) symptoms worsening

Criteria B: either of the following (a) a relative decline of ⩾10% in FVC% pred; (b) a relative decline of ⩾15% in DLCO% pred; (c) worsening symptoms and/or worsening radiological findings accompanied by ⩾5 to < 10% relative decrease in FVC% pred

When evaluating AE and OS according to univariate Cox regression analysis, predictors included phenotypic clusters (p < 0.001), diagnosis category (p < 0.001) and GAP score (p < 0.001). After adjusting for diagnosis category, corticosteroid use, immunosuppressive therapy and anti-fibrotic treatment, multivariable analysis also suggested that phenotypic clusters (p < 0.001) and GAP score (p < 0.001) independently predicted OS and AE (Table 4**)**.

**Table 4:** Variables predicting acute exacerbation and overall survival

Characteristic	Acute exacerbation			Survival		
	HR	95% CI	P	HR	95% CI	P
Univariate Cox regression						
Phenotypic cluster^a^						< 0.001
Cluster 1	2.38	1.53–3.70	< 0.001	4.06	2.10–7.86	< 0.001
Cluster 3	1.80	1.10–2.95	0.019	5.78	2.99–11.18	< 0.001
Cluster 4	0.76	0.40–1.45	0.405	1.61	0.71–3.67	0.255
Diagnosis category^b^						< 0.001
CTD-ILD	0.93	0.61–1.43	0.743	0.41	0.27–0.62	< 0.001
iNSIP	8.45	2.00-35.66	0.004	2.75	0.38–20.11	0.319
FHP	0.38	0.15–0.98	0.045	0.28	0.11–0.7	0.007
uIIP	0.89	0.43–1.84	0.756	0.29	0.12–0.74	0.010
Occupational related ILDs	0.43	0.23–0.78	0.006	0.15	0.08–0.32	< 0.001
Sarcoidosis	0.00	0	0.957	0.00	0	0.950
GAP score	1.33	1.18–1.50	< 0.001	1.47	1.29–1.69	< 0.001
Multivariable Cox regression^c^						
Phenotypic cluster^a^						< 0.001
Cluster 1	2.29	1.43–3.68	0.001	3.99	1.98–8.05	< 0.001
Cluster 3	1.21	0.65–2.27	0.546	2.90	1.31–6.41	0.008
Cluster 4	0.89	0.44–1.79	0.741	2.03	0.84–4.92	0.116
GAP score	1.31	1.16–1.48	< 0.001	1.34	1.17–1.54	< 0.001

a: Reference category: Cluster 2

b: Reference category: IPF

c: adjusted for phenotypic cluster, ILD subtype and corticosteroid use, immune-suppressive agents and anti-fibrotic treatment

## Discussion

In this retrospective cohort study, a heterogeneous group of patients with F-LID was grouped into four clusters according to clinical and comorbidities variables present before clinical diagnosis. After forming the clusters, we compared the clinical features, laboratory data, and clinical outcomes between clusters and tried to identify the group of patients with the poorest prognosis.

Cluster 1, in which 53.6% of the patients had autoimmune features, included a group of gender equally distributed patients with a higher prevalence of some common comorbidities in aging people and a higher prevalence of hypoxemia at rest, and the lung function measures at baseline were worst among four clusters. Like Cluster 1, 54.3% of patients in Cluster 2 had autoimmune features. It featured mainly young female patients with less or no smoking history. Cluster 3 included predominantly male patients with the highest rate of smoking history. The signs of velcro crackles and clubbing fingers, and the UIP pattern were presented most. Besides, we identified a cluster of patients (Cluster 4) with the highest percentage of occupational or dust exposure history. The median age for this group with the highest proportion of male patients was younger, and the clinical signs and UIP-like fibrotic pattern and emphysema on HRCT were presented least. Regarding the prognosis, Clusters 1 and 3 had a higher risk for AE, and OS was shorter in the two Clusters, consistent with clinical practice. The prevalence of fibrosis progression was also higher in Clusters 1 and 3, despite the different definitions.

Cluster analysis is a valuable approach to investigating patients with respiratory diseases [19, 20], such as asthma [21], chronic obstructive pulmonary disease [22], idiopathic pulmonary arterial hypertension [23], sarcoidosis [24], and IPF [25], as it allows the identification of distinct characteristics among the heterogeneous disease courses. A cluster analysis of patients with idiopathic interstitial pneumonia and emphysema concluded that the cluster consisting mostly of IPF patients had significantly poor mortality [26]. Another cluster analysis was performed with a cohort of 770 chronic ILD patients (37% were diagnosed with IPF) [11]. Patients with the most rapid decline in lung function, increased fibrosis progression, and poor survival were usually clustered together. And a group of elderly white male smokers with severe honeycombing had poor mortality, similar to Cluster 3 in our study. However, the study did not include patients with ILD of known etiology, of which occupational exposure is an essential factor. Our clustering was of clinical feasibility and practice in exploratory inclusion of occupational induced ILD patients. Patients in Cluster 4 in our study was characterized by non-UIP pattern on HRCT and the highest frequency of occupational exposure history. The primary underlying diagnosis for this cluster was occupational related ILDs. Generally, patients with occupational-related ILDs progress slowly, which may account for the better OS observed in this cluster [27, 28]. Conversely, Cluster 1, characterized by a high prevalence of CTD-ILD, displayed a poorer survival outcome. One plausible explanation for this observation is that patients in this cluster may have been influenced by baseline lower lung function and comorbid conditions, such as diabetes, a known risk factor for the development of IPF and vascular complications [29].

Patients with a rapid decline in lung function, increased extent of pulmonary fibrosis on chest images and worsened symptoms showed a higher risk of AE and poorer survival [2, 9]. Previously, a clinical trial reporting a beneficial effect of antifibrotic treatment in non-IPF ILDs with fibrosis progression suggested criteria B as the definition for fibrosis progression [1, 4, 30]. Recently, updated Criteria A was published [6]. Here, we adopted both two measures. In total, 26.1% of patients fulfilled Criteria A while 41.6% the Criteria B. No statistically significant difference was found in clinical features. However, the percentage of patients with AE and mortality was higher in criteria A than B, suggesting the possible sensitivity of the newly updated definition for poor prognosis.

Given the similar clinical features and prognosis of patients with PPF and IPF, the acceptance of antifibrotic treatment for IPF led to investigation of such treatment in other fibrotic lung diseases [2, 8, 31, 32]. Previously, clinical trials such as INBUILD [4], RELIEF [32] and pirfenidone on uIIP [33] reported a beneficial effect of antifibrotic treatment in non-IPF ILDs with PPF. The annual rate of decline in the FVC in patients with PF-ILD was significantly lower among patients who received nintedanib (-80.8 mL/year) than among those who received a placebo (-187.8 mL/year) [4]. In this study, OS was shorter in patients with anti-fibrotic treatment than in non-anti-fibrotic therapy in the whole cohort. After cluster and criteria stratification, no difference was found in subgroups (**Figure ****S2**). It is not unexpected that patients who received anti-fibrotic therapy would have higher mortality because the medication varied due to the compliance, socio-economic situation and medical insurance of the patients and the doctors’ prescriptions in this retrospective cohort [34]. The treatment choices could be clustered as factors in a prospective cohort in further research.

This study has several strengths. In contrast to prior studies [11, 21], we specifically included occupational induced ILD of known etiology and assessed the presence of fibrosis progression in various clusters. For occupationally related exposures such as chronic silicosis and asbestosis, the majority of patients tend to experience slow progression [27, 28]. Next, our exploration involving the inclusion of PF-ILD/PPF patients and comparison of patients meeting PF-ILD criteria to those meeting PPF criteria represented a novel and pioneering endeavor, provided valuable additional findings. Using cluster analysis, comprehensive information including demographics, physiological values, chest imaging, laboratory values and complications was provided in this study.

Several limitations should be considered. First, the monocentric and retrospective design with inherent limitations may have led to a selection and information recall bias, and the exclusion of cases with missing values during the clustering analysis undoubtedly introduces selection bias. The aims of this study and the explanations of the results should be interpreted with caution. Secondly, though we employed clinical variables that could represent patients’ demographic, historical, physical, laboratory, and radiographic information, the numbers and optimal combination of variables used for the clustering needed to be validated. A previous study has indicated that other variables like race and GERD could contribute to clustering [11], which would have provided important information regarding the outcomes of ILD. However, this was a single ethnic study and the presence of GERD was not recorded reliably in our medical system. GERD was sometimes diagnosed due to proton pump inhibitors used by patients to be eligible for government health insurance coverage. Furthermore, we compared clinical features of chronic silicosis and asbestosis patients, considering potential variations in disease progression due to distinct exposures. Our findings indicated no statistically significant differences in prognosis between these groups (**Table ****S6****and Figure ****S4**). Given the limited sample size and disease categories when stratifying disease types, a comprehensive exposure dataset is anticipated.

## Conclusion

Using baseline clinical features, our study identified four clusters of the heterogeneous group of F-ILD patients. The four clinical clusters differed in expressions of laboratory data, risks of fibrosis progression, AE, and survival, highlighting the significant heterogeneity between clusters and homogeneity within clusters and may give a clue to predict clinical prognosis and develop management strategies for these patients. Further studies are warranted to optimize the cluster variables and clinical classifications of F-ILD with prospective and multicenter study designs.

### Electronic supplementary material

Below is the link to the electronic supplementary material.

Supplementary Material 1

## Data Availability

The datasets used and/or analyzed during the current study are available from the corresponding author upon reasonable request.

## List of abbreviations

AE: acute exacerbation
ALAT: Asociación Latinoamericana de Tórax
ANA: antinuclear antibody
ATS: American Thoracic Society
BMI: body-mass index
CA125: carbohydrate antigen 125
CEA: carcinoembryonic antigen
CTD: connective tissue disease
CYFRA21-1: cytokeratin fraction 21–1
DLCO: diffusion capacity of the lung for carbon monoxide
ERS: European Respiratory Society
FHP: fibrotic hypersensitivity pneumonitis
F-ILD: fibrosing interstitial lung disease
FVC: forced vital capacity
GAP: gender-age-physiology
GERD: gastroesophageal reflux disease
HRCT: high-resolution computed tomography
ILD: interstitial lung disease
iNSIP: idiopathic non-specific interstitial pneumonia
IPF: idiopathic pulmonary fibrosis
JRS: Japanese Respiratory Society
OS: overall survival
PF-ILD: progressive fibrosing- interstitial lung disease
PPF: progressive pulmonary fibrosis
NSE: neuron-specific enolase
SCC: squamous cell carcinoma antigen
uIIP: unclassifiable idiopathic interstitial pneumonia
UIP: usual interstitial pneumonia
WBC: white blood cell
