# Supplementary material for: Cluster features in fibrosing interstitial lung disease and associations with prognosis
Source: BMC Pulm Med. 2023 Nov 1;23:420. doi: 10.1186/s12890-023-02735-7 (PMC10621076; doi:10.1186/s12890-023-02735-7)
Supplement: Supplementary file 1 — Supplementary Material 1 [file 12890_2023_2735_MOESM1_ESM.docx]

**Supplementary Material**

**Contents**

Figure S1- Flow chart of enrolled patients 2

Figure S2- Overall survival of anti-fibrotic treatment versus non-anti-fibrotic treatment 3

Figure S3- Predictor importance 4

Figure S4- Overall survival and acute exacerbation of chronic silicosis versus asbestosis 5

Table S1- Demographics and clinical characteristics of patients with fibrosis progression 6

Table S2- The expressions of blood cell counts, the derivative inflammation indexes, and serum oncomarkers of the whole cohort 8

Table S3- The expressions of routine blood counts, the derivative inflammation indexes, and serum oncomarkers of patients with fibrosis progression 9

Table S4- Auto-Clustering 10

Table S5. Demographics and clinical characteristics of the patients with missing data

11

Table S6. Demographics and clinical characteristics of the patients with occupational

related ILDs

12


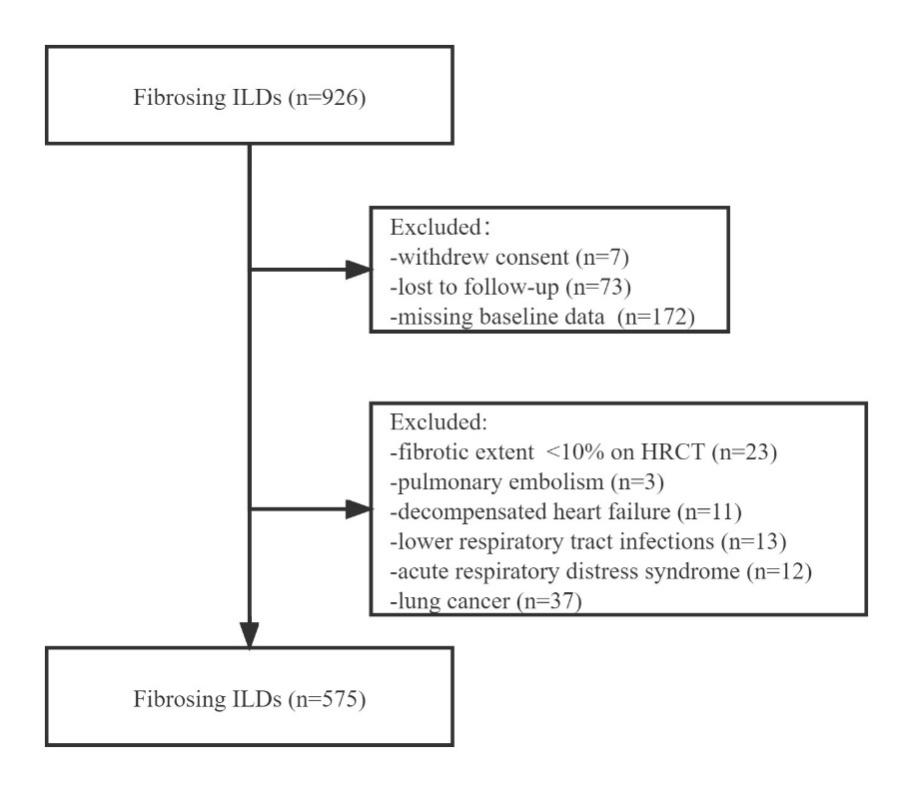


Figure S1. Flow chart of enrolled patients

Abbreviations: HRCT: high-resolution computed tomography; ILD: interstitial lung disease


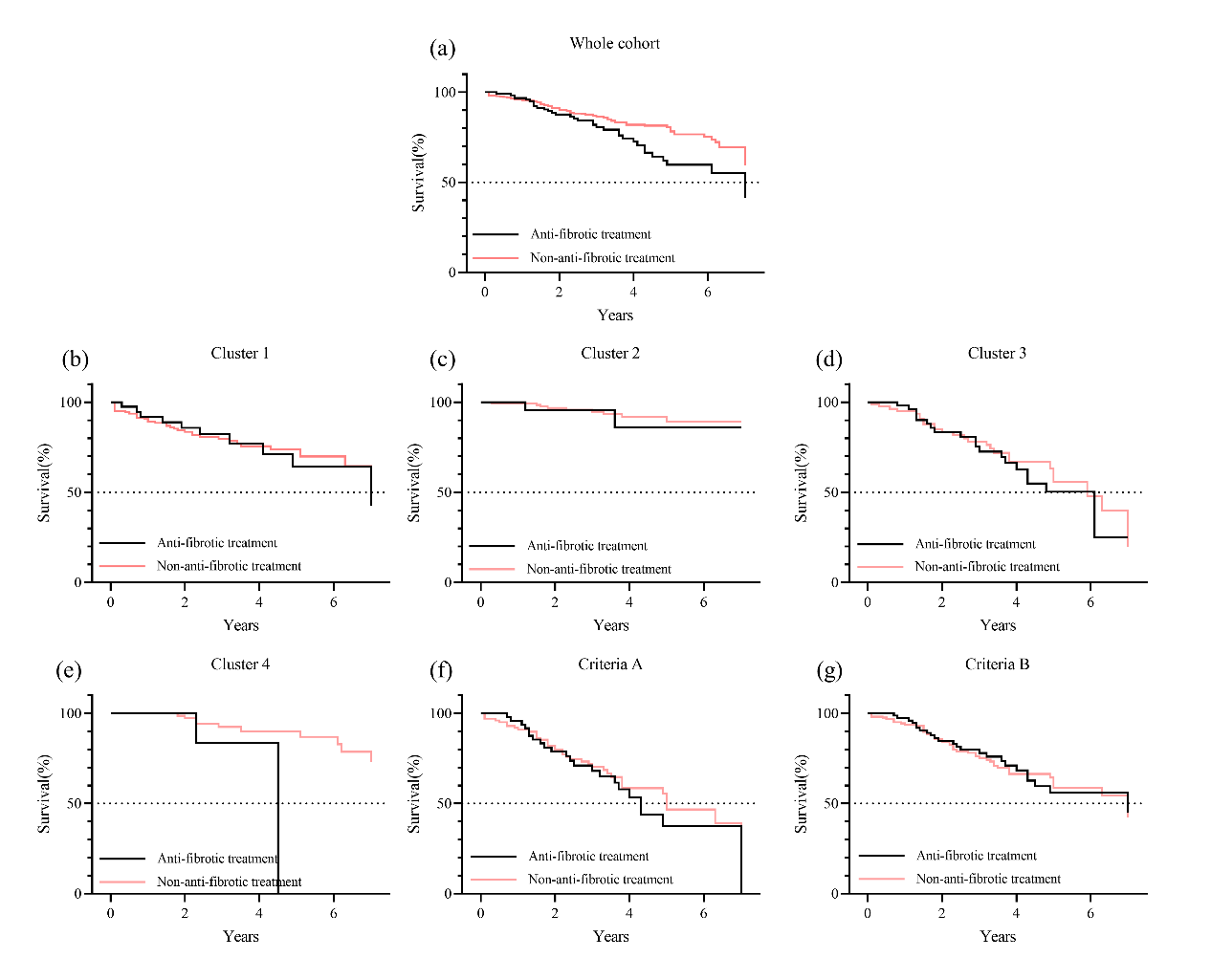
Figure S2. (a) OS was significantly shorter in patients with anti-fibrotic treatment (black line, median OS =7.0 years) than in non-anti-fibrotic treatment (pink line, median OS=not reached, p=0.017). (b) no difference was observed in Cluster 1 (p=0.927). (c) no difference was observed in Cluster 2 (p=0.759). (d) no difference was observed in Cluster 3 (p=0.610). (e) no difference was observed in Cluster 4 (p=0.067). (f) no difference was observed in fibrosis progression patients with criteria A (p=0.482). (g) no difference was observed in fibrosis progression patients with criteria B (p=0.918).


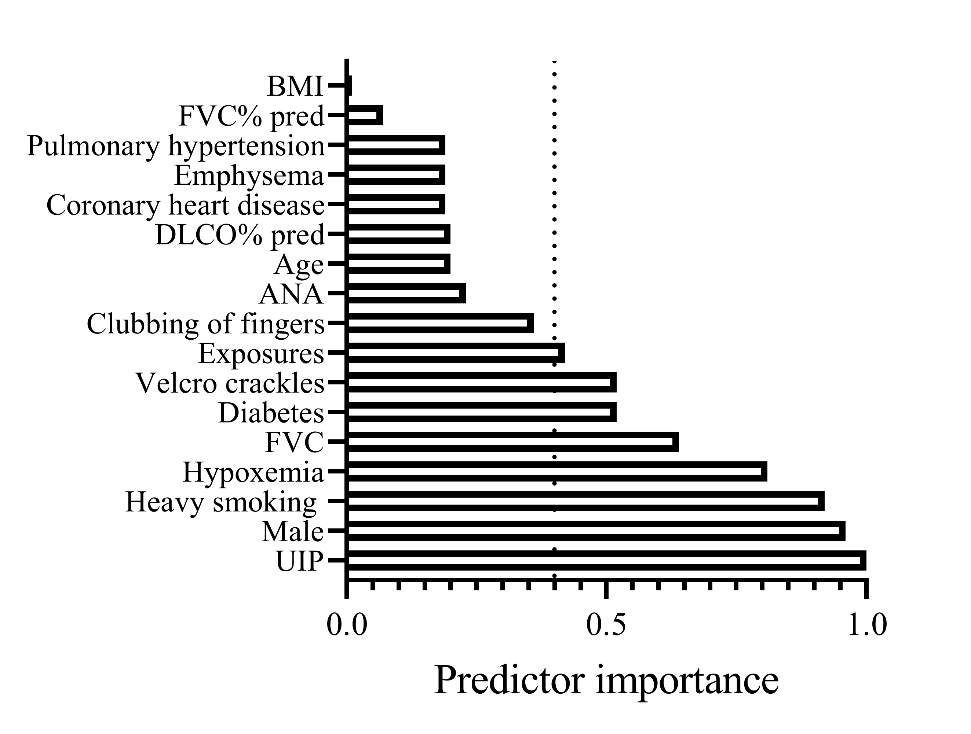


Figure S3. Predictor importance

Abbreviations: ANA: antinuclear antibody; BMI: body-mass index; DLCO: diffusion capacity of the lung for carbon monoxide; FVC: forced vital capacity; HRCT: high-resolution computed tomography; UIP: usual interstitial pneumonia


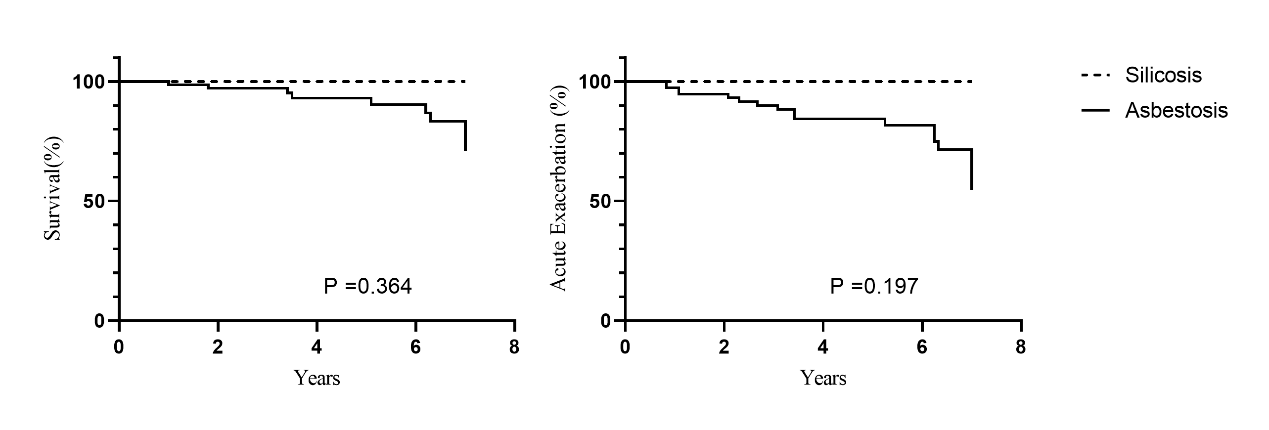


Figure S4. Overall survival and acute exacerbation of chronic silicosis versus asbestosis

Table S1. Demographics and clinical characteristics of patients with fibrosis progression

|  | | Criteria A,  n=150 | Criteria B  n=239 | *P-*value |
| --- | --- | --- | --- | --- |
| Age (quartile) | | 65 (59.75,73) | 65 (57,72) | 0.421 |
| Male, n (%) | | 75 (50) | 118 (49.4) | 0.904 |
| BMI (quartile) | | 25.9 (24.1,28.0) | 25.8 (23.4,27.9) | 0.471 |
| Smoking status, n (%) | |  |  |  |
|  | Ever smokers | 57 (38) | 88 (36.8) | 0.815 |
|  | Heavy smokers | 47 (31.3) | 63 (26.4) | 0.289 |
|  | Smoking cessation >1 year | 46 (64.8) | 70 (68) | 0.663 |
| Exposure history, n (%) | | 62 (41.3) | 100 (41.8) | 0.921 |
|  | Inorganic | 32 (21.3) | 48 (20.1) |  |
|  | Organic | 25 (16.7) | 43 (18) |  |
|  | Mixed | 3 (2) | 7 (2.9) | 0.922 |
| Symptoms, n (%) | |  |  |  |
|  | Dry cough | 102 (85) | 153 (82.7) | 0.597 |
|  | Productive cough | 28 (18.7) | 43 (18) | 0.867 |
|  | Shortness of breath | 85 (56.7) | 143 (59.8) | 0.537 |
|  | Joint discomfort | 21 (14) | 36 (15.1) | 0.773 |
| Signs, n (%) | |  |  |  |
|  | Clubbing of fingers | 47 (31.3) | 81 (33.9) | 0.601 |
|  | Velcro crackles | 121 (80.7) | 197 (82.4) | 0.662 |
| Pulmonary function | |  |  |  |
|  | FVC (L) (SD) | 2.5 (0.8) | 2.5 (0.8) | 0.908 |
|  | FVC% pred (quartile) | 83.9 (72.8,98.5) | 83.7 (71.8,97.6) | 0.776 |
|  | DLCO% pred (quartile) | 58.4 (46.8,70.8) | 57.9 (46.7,70.7) | 0.990 |
| HRCT, n (%) | |  |  |  |
|  | UIP-like pattern | 54 (36) | 75 (31.4) | 0.346 |
|  | Emphysema | 9 (6) | 16 (6.7) | 0.786 |
| Hypoxemia at rest, n (%) | | 58 (38.7) | 85 (35.6) | 0.537 |
| ANA, n (%) | | 59 (39.3) | 99 (41.4) | 0.683 |
| Comorbidities, n (%) | |  |  |  |
|  | Pulmonary hypertension | 22 (14.7) | 34 (14.2) | 0.904 |
|  | Diabetes | 46 (30.7) | 74 (31) | 0.951 |
|  | Coronary heart disease | 26 (17.3) | 38 (15.9) | 0.71 |
|  | Hypertension | 62 (41.3) | 91 (38.1) | 0.522 |
|  | Hypothyroidism | 6 (4) | 9 (3.8) | 0.907 |
| Treatment, n (%) | |  |  |  |
|  | Corticosteroid | 85 (56.7) | 148 (61.9) | 0.303 |
|  | Immunosuppressive agents | 54 (36) | 105 (43.9) | 0.121 |
|  | Anti-fibrotic treatment | 49 (32.7) | 75 (31.4) | 0.791 |
|  | Long-term oxygen therapy | 25 (16.7) | 37 (15.5) | 0.756 |
| AE, n (%) | | 66 (44) | 92 (38.5) | 0.282 |
| Death, n (%) | | 61 (40.7) | 74 (31) | 0.05 |

Data were presented as the mean (SD), or median (quartile), or numbers (%).

*: Heavy smokers: smoking index = daily tobacco intake × duration of smoking≥400

Abbreviations: ANA: antinuclear antibody; BMI: body-mass index; DLCO: diffusion capacity of the lung for carbon monoxide; FVC: forced vital capacity; HRCT: high-resolution computed tomography; UIP: usual interstitial pneumonia

Table S2. Blood cell counts, the derivative inflammation indexes, and serum oncomarkers of the whole cohort

|  | Cluster 1  n=181 | Cluster 2  n=164 | Cluster 3  n=134 | Cluster 4  n=96 | All  n=575 | *P-*value |
| --- | --- | --- | --- | --- | --- | --- |
| Routine blood counts |  |  |  |  |  |  |
| WBC (n*10^9^/L) | 8.12 (3.54)^†^ | 6.69 (2.46)^‡§^ | 7.73 (2.82) | 7.55 (2.88) | 7.52 (3.03) | <0.001 |
| Neutrophil (*10^9^/L) | 5.66 (3.28)^†^ | 4.33 (1.95)^§^ | 4.90 (2.49) | 5.4 1(6.67) | 5.06 (3.68) | 0.007 |
| Lymphocyte (*10^9^/L) | 1.68 (0.83)^‡§^ | 1.67 (0.71)^‡§^ | 2.06 (0.78) | 2.28 (2.21) | 1.86 (1.17) | <0.001 |
| Monocyte (*10^9^/L) | 0.48 (0.29) | 0.43 (0.18)^‡§^ | 0.54 (0.40) | 0.52 (0.25) | 0.49 (0.29) | 0.005 |
| Hemoglobin (g/L) | 126.29 (17.98)^‡§^ | 124.97 (20.02)^‡§^ | 141.13 (24.61) | 140.44 (18.12) | 131.73 (21.58) | <0.001 |
| RDW-CV (%) | 13.58 (1.30)^‡^ | 13.46 (1.91)^‡^ | 15.85 (17.94)^§^ | 13.09 (1.09) | 13.99 (8.80) | 0.046 |
| Platelet (*10^9^/L) | 241.68 (88.16)^‡^ | 237.82 (69.54) | 221.47 (84.62) | 226.47 (65.67) | 233.33 (79.07) | 0.098 |
| Inflammation Indexes |  |  |  |  |  |  |
| MLR | 4.21 (2.78)^§^ | 4.2 (1.89)^§^ | 4.47 (2.07) | 4.86 (4.56) | 4.38 (2.81) | 0.242 |
| NLR | 4.85 (6.40)^†‡§^ | 3.31 (3.27) | 2.86 (2.17) | 2.74 (2.00) | 3.59 (4.29) | <0.001 |
| PLR | 180.73 (120.26)^‡§^ | 180.07 (131.95)^‡§^ | 129.3 (103.46) | 122.65 (53.14) | 158.86 (114.58) | <0.001 |
| Oncomarkers |  |  |  |  |  |  |
| SCC | 1.34 (2.82)^†^ | 0.91 (0.96) | 1.25 (1.42) | 1.11 (0.98) | 1.16 (1.85) | 0.163 |
| CEA | 3.37 (4.20)^‡§^ | 2.21 (3.65)^‡^ | 3.90 (3.80)^§^ | 2.44 (1.93) | 3.01 (3.70) | <0.001 |
| CYFRA21-1 | 5.63 (7.96)^§^ | 4.84 (8.39)^§^ | 4.74 (5.78)^§^ | 2.87 (1.84) | 4.74 (7.00) | 0.02 |
| CA125 | 26.43 (28.97)^§^ | 16.54 (16.34)^‡^ | 29.90 (30.99)^§^ | 19.70 (24.81) | 23.30 (26.31) | <0.001 |
| NSE | 17.23 (8.37)^†‡§^ | 15.51(6.86) | 15.29 (6.45) | 14.70 (4.19) | 15.86 (6.98) | 0.012 |

Data are presented as the mean (SD).

†: p<0.05 compared with Cluster 2. ‡: p<0.05 compared with Cluster 3. §: p<0.05 compared with Cluster 4.

Abbreviations: WBC: white blood cell; RDW-CV: red cell distribution width (coefficient of variation); NLR: neutrophil-to-lymphocyte ratio; MLR: monocyte-to-lymphocyte ratio; PLR: platelet-to-lymphocyte ratio; SCC: squamous cell carcinoma antigen; CEA: carcinoembryonic antigen; CYFRA21-1: cytokeratin fraction 21–1; CA125: carbohydrate antigen 125; NSE: neuron-specific enolase.

Table S3. Blood cell counts, the derivative inflammation indexes, and serum oncomarkers of patients with fibrosis progression

|  | Criteria A,  n=150 | Criteria B  n=239 | *P*-value |
| --- | --- | --- | --- |
| Routine blood counts |  |  |  |
| WBC (n*10^9^/L) | 7.82 (3.01) | 7.61 (2.83) | 0.47 |
| Neutrophil (*10^9^/L) | 5.16 (2.54) | 4.98 (2.43) | 0.49 |
| Lymphocyte (*10^9^/L) | 1.83 (0.88) | 1.84 (0.87) | 0.934 |
| Monocyte (*10^9^/L) | 0.51 (0.30) | 0.51 (0.33) | 0.985 |
| Hemoglobin (g/L) | 132.73 (25.62) | 132.41 (23.19) | 0.897 |
| RDW-CV (%) | 15.76 (16.96) | 14.9 (13.50) | 0.577 |
| Platelet (*10^9^/L) | 226.25(85.10) | 225.1 (78.18) | 0.892 |
| Inflammation Indexes |  |  |  |
| MLR | 4.13 (2.08) | 4.13 (2.00) | 1 |
| NLR | 3.63 (3.34) | 3.47 (3.16) | 0.626 |
| PLR | 153.81 (113.32) | 150.87 (103.78) | 0.793 |
| Oncomarkers |  |  |  |
| SCC | 1.49 (2.95) | 1.36 (2.51) | 0.651 |
| CEA | 3.57 (4.37) | 3.34 (3.93) | 0.591 |
| CYFRA21-1 | 5.9 (10.15) | 5.36 (8.60) | 0.576 |
| CA125 | 26.69 (31.62) | 25.58 (29.13) | 0.723 |
| NSE | 15.71 (8.53） | 15.53 (7.24) | 0.825 |

Data are presented as the mean (SD).

Abbreviations: WBC: white blood cell; RDW-CV: red cell distribution width (coefficient of variation); NLR: neutrophil-to-lymphocyte ratio; MLR: monocyte-to-lymphocyte ratio; PLR: platelet-to-lymphocyte ratio; SCC: squamous cell carcinoma antigen; CEA: carcinoembryonic antigen; CYFRA21-1: cytokeratin fraction 21–1; CA125: carbohydrate antigen 125; NSE: neuron-specific enolase.

| Table S4. Auto-Clustering |
| --- |

| Number of Clusters | Schwarz's Bayesian Criterion (BIC) | BIC Change^a^ | Ratio of BIC Changes^b^ | Ratio of Distance Measures^c^ |
| --- | --- | --- | --- | --- |
| 1 | 1702.602 |  |  |  |
| 2 | 1436.618 | -265.984 | 1.000 | 1.824 |
| 3 | 1327.583 | -109.035 | .410 | 1.238 |
| 4 | 1239.214 | -72.369 | .272 | 1.674 |
| 5 | 1244.733 | -10.481 | .039 | 1.045 |
| 6 | 1238.235 | -6.498 | .024 | 1.104 |
| 7 | 1240.037 | 1.801 | -.007 | 1.170 |

| 1. The changes are from the previous number of clusters in the table. |
| --- |
| 1. The ratios of changes are relative to the change for the two cluster solution. |
| 1. The ratios of distance measures are based on the current number of clusters against the previous number of clusters. |

Table S5. Demographics and clinical characteristics of the patients with missing data

|  | | Missing data,  n=172 | All,  n=575 | P |
| --- | --- | --- | --- | --- |
| Age (quartile) | | 63(55,68, n=139) | 63 (56,70) | 0.173 |
| Male, n (%) | | 76 (46.9, n=162) | 293 (51.0) | 0.363 |
| BMI (quartile) | | 26.0 (24.2,27.5, n=138) | 25.9 (23.9,27.9) | 0.719 |
| Ever smokers, n (%) | | 54 (34.8, n=155) | 218 (37.9) | 0.051 |
| Exposure history, n (%) | | 47 (30.5, n=154) | 241 (41.9) | 0.01 |
|  | Inorganic | 9 (5.8) | 129 (22.4) |  |
|  | Organic | 31 (20.1) | 92 (16.0) |  |
|  | Mixed | 7 (4.5) | 16 (2.8) | <0.001 |
| Pulmonary function | |  |  |  |
|  | FVC (L) (SD) | 2.49 (0.8, n=144) | 2.6 (0.8) | 0.378 |
|  | FVC% pred (quartile) | 83.5 (70.6,100.4, n=138) | 85.2 (72,100.4) | 0.756 |
|  | DLCO% pred (quartile) | 60.3 (46.3,73.1, n=138) | 60.3 (48.2,74) | 0.347 |
| HRCT, n (%) | |  |  |  |
|  | UIP-like pattern | 44 (26.5, n=172) | 137 (23.8) | 0.637 |
|  | Emphysema | 18 (10.5, n=172) | 35 (6.1) | 0.05 |
| Treatment, n (%) | |  |  |  |
|  | Corticosteroid | 111 (72.1, n=154) | 358 (62.3) | 0.024 |
|  | Immunosuppressive agents | 78 (52, n=150) | 251 (43.7) | 0.067 |
|  | Anti-fibrotic treatment | 29 (20.6, n=141) | 122 (21.2) | 0.865 |
|  | Long-term oxygen therapy | 10 (6.3, n=158) | 71 (12.3) | 0.033 |
| Death, n (%) | | 38 (22.6, n=168) | 113 (19.7) | 0.401 |

Data were presented as the mean (SD), median (quartile), or numbers (%).

*: Heavy smokers: smoking index = daily tobacco intake × duration of smoking≥400

Abbreviations: BMI: body-mass index; DLCO: diffusion capacity of the lung for carbon monoxide; FVC: forced vital capacity; HRCT: high-resolution computed tomography; SD: standard deviation; UIP: usual interstitial pneumonia

Table S6. Demographics and clinical characteristics of the patients with occupational

related ILDs

|  | | Chronic silicosis,  n=17 | Asbestos,  n=76 | P |
| --- | --- | --- | --- | --- |
| Age (quartile) | | 63.5(56.5,68) | 67.5 (62.5,72.5) | 0.005 |
| Male, n (%) | | 14 (82.4) | 41 (53.9) | 0.031 |
| BMI (quartile) | | 25.6 (23.5,26.8) | 27.1 (24.5,29.1) | 0.081 |
| Smoking status, n (%) | |  |  |  |
|  | Ever smokers | 11 (64.7) | 30 (39.5) | 0.058 |
|  | #Heavy smokers* | 10 (58.8) | 16 (21.1) | 0.002 |
|  | Smoking cessation >1 yr | 8 (47.1) | 16 (21.1) | 0.027 |
| Pulmonary function | |  |  |  |
|  | FVC (L) (SD) | 3.1 (0.9) | 2.2 (0.75) | 0.001 |
|  | FVC% pred (quartile) | 98.1 (91.1,110) | 78.4 (60.9,92.9) | 0.002 |
|  | DLCO% pred (quartile) | 84.1 (56.6, 102.7) | 70.3 (57.2,85.2) | 0.021 |
| HRCT, n (%) | |  |  |  |
|  | Honeycombing | 0 | 10 (13.2) | 0.113 |
|  | Emphysema | 1 (5.9) | 3 (3.9) | 0.722 |
| GAP- Score | | 2 (1.3) | 3 (3,4) | 0.026 |
| Fibrosis progression, n (%) | |  |  |  |
|  | Criteria A (1 year) | 3 (17.6) | 22 (28.9) | 0.388 |
|  | Criteria B (2 years) | 5 (29.4) | 22 (28.9) | 0.97 |
| Acute exacerbation, n (%) | | 0 | 18 (23.7) | 0.025 |
| Death, n (%) | | 0 | 10 (13.2) | 0.113 |

Data were presented as the mean (SD), median (quartile), or numbers (%).

*: Heavy smokers: smoking index = daily tobacco intake × duration of smoking≥400

Abbreviations: BMI: body-mass index; DLCO: diffusion capacity of the lung for carbon monoxide; FVC: forced vital capacity; GAP: gender-age-physiology; HRCT: high-resolution computed tomography; SD: standard deviation
